# Mindful Parenting Training in Child Psychiatric Settings: Heightened Parental Mindfulness Reduces Parents’ and Children’s Psychopathology

**DOI:** 10.1007/s12671-016-0504-1

**Published:** 2016-03-15

**Authors:** Renée Meppelink, Esther I. de Bruin, Femy H. Wanders-Mulder, Corinne J. Vennik, Susan M. Bögels

**Affiliations:** Research Institute of Child Development and Education, University of Amsterdam, Nieuwe Achtergracht 127, Amsterdam, 1018 WS The Netherlands; Research Priority Area Yield, University of Amsterdam, Amsterdam, The Netherlands; Accare, Center for Child and Adolescent Psychiatry, Groningen, The Netherlands; Jeugd GGZ/Dimence, Outpatient Mental Health Care Center, Zutphen, The Netherlands; UvA minds, Academic Outpatient Child and Adolescent Treatment Center, Amsterdam, The Netherlands

**Keywords:** Mindful parenting, Child psychopathology, Parental psychopathology

## Abstract

Mindful parenting training is an application of mindfulness-based interventions that allows parents to perceive their children with unbiased and open attention without prejudgment and become more attentive and less reactive in their parenting. This study examined the effectiveness of mindful parenting training in a clinical setting on child and parental psychopathology and of mindfulness as a predictor of these outcomes. Seventy parents of 70 children (mean age = 8.7) who were referred to a mental health care clinic because of their children’s psychopathology participated in an 8-week mindful parenting training. Parents completed questionnaires at pre-test, post-test and 8-week follow-up. A significant decrease was found in children’s and parents’ psychopathology and a significant increase in mindful parenting and in general mindful awareness. Improvement in general mindful awareness, but not mindful parenting, was found to predict a reduction in parental psychopathology, whereas improvement in mindful parenting, but not general mindful awareness, predicted the reduction of child psychopathology. This study adds to the emerging body of evidence indicating that mindful parenting training is effective for parents themselves and, indirectly, for their children suffering from psychopathology. As parents’ increased mindful parenting, but not increased general mindfulness, is found to predict child psychopathology, mindful parenting training rather than general mindfulness training appears to be the training of choice. However, RCTs comparing mindful parenting to general mindfulness training and to parent management training are needed in order to shed more light on the effects of mindful parenting and mechanisms of change.

## Introduction

Mindful parenting is an application of mindfulness-based interventions. Bringing mindfulness principles into parenting allows parents to perceive their children with unbiased and open attention without prejudgment, allowing for more sensitive and responsive reactions to their children’s needs and behaviour, instead of reacting automatically (Kabat-Zinn and Kabat-Zinn 1998). Being a parent is a challenging, time consuming, and responsible task. Although parents generally raise their child with dedication and love, they commonly simultaneously experience stress. Parenting may be further complicated when the parent or the child have psychopathology symptoms, such as a depressed mood, anxiety, attention and behavioural problems (Bögels and Restifo 2014). Several behavioural parent programs have been developed to support parents in the upbringing of a child with psychopathology symptoms, by teaching them behavioural strategies to cope with the problem behaviour of the child. However, children from parents suffering from psychopathology symptoms themselves, such as depression or ADHD, are less likely to benefit from these parent training programs (e.g. Reyno and McGrath 2006; Sonuga-Barke et al. 2002). A mindful parenting training takes another approach as it aims to heighten parents’ awareness of their own stress and psychopathology symptoms in reaction to their child’s behaviour. Parents are invited to take a more accepting and less reactive attitude toward their child’s and their own (behavioural) problems, and to take better care of themselves, which in turn may improve the problem behaviour of the child (Bögels and Restifo 2014).

Mindful parenting training as a clinical intervention is relatively new. Five clinical trials and three single-case studies have been conducted. In the currently largest study (Dykens et al. 2014) on the effects of a mindfulness-based intervention for parents, mothers (*N* = 243) of children with autism (65 %) or other disabilities (35 %) were randomised into a 6-week training for either mindfulness-based stress reduction (MBSR) or positive adult development (PAD). Participants were assessed at baseline, during the training, directly after the training, and at three follow-up assessments. Both arms showed a significant decline in stress, depression and anxiety, and improved sleep and life satisfaction. Furthermore, mothers receiving MBSR showed larger reductions in anxiety and depression, and larger improvements in sleep and well-being, compared to mothers in the PAD training. Neece (2014) randomly assigned 46 parents of children (aged 2.5–5) with developmental delays to an 8-week MBSR group or a waitlist control group and completed a pre- and a post-test. Parents in the MBSR group were found to have significantly less stress and depression and greater life satisfaction as compared to the waitlist control group. In addition, parents in the MBSR group reported a marginally significant reduction in children’s attention problems and a significant reduction in attention-deficit/hyperactivity disorder (ADHD) symptoms. It has to be noted, however, that the two above mentioned studies (Dykens et al. 2014; Neece 2014) evaluated the effects of a MBSR training and not a mindful parenting training that specifically incorporates mindfulness, self-awareness, and intentionality into the parent–child relationship. However, Neece (2014) did find reductions in children’s psychopathology.

Ferraioli and Harris (2013) compared an 8-week mindfulness-based parent training to an 8-week skills-based parent training using a pre-post-follow-up design. Fifteen parents of children with autism were randomly allocated to one of the two treatment groups. The mindfulness group, but not the skills group, demonstrated a significant decrease on parental stress and a significant increase on global health and mindful awareness in daily life following treatment. Srivastava et al. (2011) evaluated effects of a 24-session mindful parenting training using a single group pre- and post-research design, in parents of 60 children (aged 3–6) referred to paediatric care because of various behavioural problems. The severity of disturbed behaviour in the children declined after training, as did hostile/aggressive behaviour, anxious behaviour and hyperactive/distractible behaviour. Bögels et al. (2014) evaluated the effects of an 8-week mindful parenting training using a pre-post-follow-up design with a waitlist control group. Parents (*N* = 86) were referred to an outpatient mental health care clinic because of their children’s and/or their own psychiatric symptoms and/or parent-child relationship problems. Findings indicated a reduction in both children’s and parents’ internalising and externalising psychopathology of small to medium effect size. Improvements were also found on measures of parenting and co-parenting, and reductions were found in parental stress. These results were mostly maintained at the 8-week follow-up.

Next to these five larger trials in clinical populations, three case studies were conducted by Singh and colleagues (2006, 2007, 2014) evaluating the effects of mindful parenting training for mothers of children with autism spectrum disorder (2006, 2014) and developmental problems (2007). Individual single-subject designs in a mental health care setting were used; per study, three (2006, 2014) or four (2007) mothers participated. Generally, results show a decrease in their child’s aggression, non-compliance, and self-injury, and decreased parenting stress. Furthermore, an enhancement was reported in (satisfaction with) their own parenting skills and social interaction with their children.

From the studies discussed above, we can conclude that mindfulness-based therapies for parents have a positive effect on various parent and child variables. However, only Neece (2014) and Bögels et al. (2014) assessed parents’ own psychopathology symptoms as well their children’s, while in a clinical sample, these are relevant variables to take into account. Moreover, only Ferraioli and Harris (2013) included measures on mindfulness or mindful parenting, while improved mindfulness or mindful parenting is thought of as an underlying mechanism of change.

A meta-analytic review of on the mechanisms of mindfulness-based interventions, such as MBSR and mindfulness-based cognitive therapy (MBCT), was conducted by Gu et al. (2015). Twelve randomised controlled trails and four quasi-experimental studies were evaluated, of which 13 included both mindfulness as a mediator and mental health as the outcome variable (i.e., depression, stress, anxiety, mood states, quality of life and anger expression). Findings of the narrative synthesis and the quantitative synthesis, using two-stage meta-analytic structural equation modelling, showed consistent evidence for mindfulness as a mechanism of change on the aforementioned mental health outcomes. Mindfulness-based interventions are thought of as a mean to develop skills, such as open awareness towards thoughts, emotions, and physical sensations, acceptance and compassion towards oneself and others, and a non-judgemental and non-reactive attitude towards everyday experiences, which in turn may positively affect psychopathological outcomes in individuals (Segal et al. 2012). Parents in mindful parenting training are taught to apply these skills in the interaction with their child and in difficult parenting situations, which may indirectly improve psychopathology outcomes in their children as well. Therefore, both general mindful awareness and mindful parenting are possible mechanisms of change in mindful parenting training. Exploring these potential mechanisms of change in the light of psychological outcomes in parents and children will contribute to the current knowledge of why and how mindful parenting training works. Knowing what aspects of the training work is also important for improving the efficacy of this new training for parents.

The aim of this study is to contribute to the current understanding of the direct and delayed effects of a single mindful parenting training in a clinical setting on child and parental psychopathology and of mindfulness as a predictor of these outcomes. We hypothesise that (1) psychopathology in both parents and children decreases after parents’ participation in mindful parenting training and that (2) mindful parenting and general mindful awareness in parents increases after parents’ participation in mindful parenting training. Both hypotheses are a precondition for the hypothesis (3) that the effects of the training on parents’ general mindful awareness and mindful parenting predict a decline in child and parental psychopathology.

## Method

### Participants

In this study, 70 parents (*M*_age_ = 42.0; *SD* = 7.2; 65 mothers and 5 fathers) of 70 children (*M*_age_ = 8.7; *SD* = 3.4; 40 boys and 30 girls) participated. Two husbands of two participating mothers also participated in the mindful parenting training and filled out questionnaires; however, because the dependency of this data, fathers’ data were not included into the analyses. Three outpatient mental health care clinics participated in this study, UvA minds (*N* = 34), Dimence (*N* = 23), and Accare (*N* = 13), to which parents were referred because of their child’s psychopathology by their general practitioner. In consultation with the psychiatrist of the treatment centre, parents were given the opportunity to participate in the mindful parenting training. Once they decided to participate in the training, they were asked by a researcher to participate in the study. One child mental health care clinic (UvA minds) is situated in an urban area in The Netherlands, and the other two (Dimence and Accare) in a more rural area of which one (Accare) is an infant mental health care setting.

Children were classified according to the guidelines of the Diagnostic and Statistical Manual of Mental Disorders (DSM-IV; American Psychiatric Association 2000); 28.6 % of the children were diagnosed with autism spectrum disorder, 24.3 % with ADHD, 2.9 % with an anxiety disorder, 1.4 % with oppositional defiant disorder, 1.4 % with adjustment disorder, and 25.7 % had a V-code parent–child interaction problem. Some children (5.7 %) were classified with other DSM-IV diagnoses. Two children (2.9 %) did not have a diagnosis, but their parents were treated for their own psychopathology, as they were diagnosed with adjustment disorder. Moreover, the other children (7.1 %) did not meet the threshold for a DSM-IV classification, but psychopathology symptoms were present.

Inclusion criteria were having a child with a DSM-IV classification or serious psychiatric problems. Parents were excluded when they had an estimated IQ < 80 (based on clinical dossiers, which usually included results of an IQ test), inadequate mastery of the Dutch language, or when parent(s) or child(ren) participated in another parallel on-going psychological intervention. The majority of the parents were born in the Netherlands (80 %); other parents were born in the UK (5.7 %), the Antilles (2.9 %), Germany (1.4 %) and Surinam (1.4 %). Of 8.6 % of the parents, the country of birth was not known. The median of parents’ educational level was intermediate vocational education (30 %), which was coded as a 5 on a scale from 1 = primary school to 7 = university college. The median of parents’ professional level was a full-time job, accounting for 30 % of the parents, and 18.6 % were unemployed. The other parents were housewives/housemen (11.4 %), working part-time (2.9 %), or referred themselves as ‘other’ (8.6 %). Of 28.6 % of the parents, the professional level was unknown. Participants differed per location on age (*F*(2,67) = 6.1, *p* < .01), as participants of Accare were on average younger than those of UvA minds and Dimence. Furthermore, participants differed in educational level (*F*(2,56) = 4.8, *p* < .05), as participants of UvA minds were on average higher educated than those of Dimence and Accare. Parents did not differ in ethical background nor in professional level.

In total, ten groups were conducted, four at UvA minds (groups ranging from 6 to 13 participants), three at Dimence (with 8 participants in each group) and three at Accare (with 4 to 5 participants in each group). Three mothers (4.3 %) dropped out of the training before the end and did not complete further assessments. Furthermore, three mothers (4.3 %) who did complete the training stopped participating in the study at post-test and six mothers (8.6 %) at follow-up.

### Procedure

A quasi-experimental design was used. The week before treatment started, pre-test took place. Immediately after the 8-week course, a post-test was conducted and 8 weeks later parents filled out follow-up assessment. All questionnaires were completed at home.

The mindful parenting training is an 8-week course in group format, consisting of 3-h weekly sessions and at least 1-h of meditation practice a day, following the manual as described in Bögels et al. (2014), and more extensively in Bögels and Restifo (2014), but note that the formal loving kindness and self-compassion practices that are described in Bögels and Restifo (2014) were not yet part of this manual. Children were not involved in the training. About two thirds of each session focussed on regular MBSR/MBCT meditation practices such as the body scan, sitting meditation, breathing space, seeing and hearing meditation, mindful walking and yoga. Roughly one third of each session consisted of mindful parenting principles: understanding the effect of parental reactivity, taking care of oneself as a parent, non-judgmental and open attention to one’s child, acceptance of the child and his or her difficulties, and reducing parental stress (Bögels and Restifo 2014). As children of the parents participating in this training formed a heterogeneous group, the program focused mainly on parenting under stress, and on parenting children with a large variety of mental health problems.

Homework consisted not only of meditation exercises; parents were also encouraged to apply mindfulness to interactions with their children. Parents received a CD with mindfulness meditation exercises at the beginning of the training together with a binder with instructions for home practice and session handouts. Mindful parenting trainers were mental health professionals working with children and parents; all had received basic training in mindfulness and followed an 8-day advanced teacher training in mindful parenting led by Susan Bögels and Joke Hellemans, before running their own mindful parenting groups.

### Measures

#### Mindful Parenting

Parents were asked to fill out the Dutch version of the original short version of the Interpersonal Mindfulness in Parenting scale (IM-P; De Bruin et al. 2014; Duncan 2007) at pre-test, post-test and follow-up. The short version of IM-P is a ten-item questionnaire, rated on a five-point Likert scale ranging from 1 (never true) to 5 (always true) and is designed to measure mindful parenting in four subscales: (1) Present-Centred Attention in Parenting (two items), (2) Present-Centred Emotional Awareness in Parenting (two items), (3) Non-Reactivity/Low-Reactivity in Parenting (three items), and (4) Non-Judgmental Acceptance in Parenting (three items). An example question is, ‘When I’m upset with my child, I notice how I am feeling before I take action’. Internal reliability for this questionnaire was found to be acceptable with a Cronbach’s alpha of .72 (Duncan 2007). In this study, reliability was found to be insufficient at pre-test and satisfactory at post-test and follow-up, respectively, Cronbach’s alpha was .54, .72, and .72. The removal of item 3 (‘I notice how changes in my child’s mood affect my mood’) was found to improve reliability for all three measurement occasions, yielding a Cronbach’s alpha at pre-test, post-test and follow-up of respectively .62, .75 and .75. In all further analyses, item 3 of the IM-P was not included.

#### General Mindful Awareness

To assess general mindful awareness in parents, the short 24-item Dutch version of the Five Facet Mindfulness Questionnaire (FFMQ; Baer et al. 2006; De Bruin et al. 2012) was used. The FFMQ is rated on a five-point Likert scale from 1 (never or very rarely true) to 5 (very often or always true) and measures five domains of mindful awareness. The first domain is Observing, referring to the competence to notice or attend to internal and external experiences, such as emotions, thoughts and sensory perceptions. The second is Describing, referring to the ability to describe and label these experiences verbally. Acting with awareness is the third, measuring the ability to bringing full awareness to current activity or experiences. The fourth is Non-judging, measuring non-evaluative attitude towards inner experiences. Finally, Non-reactivity is the fifth domain, referring to the competence to allow mental processes and feelings to come and go, without getting carried away by it. Combining the five facets of this questionnaire, a total score is obtained, reflecting a measure of general mindfulness (De Bruin et al. 2012; Williams et al. 2014). An example question is ‘I can usually describe how I feel at the moment in considerable detail’. In this study, internal reliability was found to be good at pre-test, post-test and follow-up; Cronbach’s alpha was respectively .84, .86 and .90.

#### Parent’s and Child’s Psychopathology

The Dutch version of the Adult Self Report (ASR; Achenbach and Rescorla 2003) and the Dutch version of the Child Behavior Checklist for both the ages 1.5–5 and 6–18 (CBCL; Achenbach 1991; Achenbach and Rescorla 2000) were used to assess parent’s and child’s psychopathology symptoms respectively. The ASR consists of 126 items, whereas the CBCL consists of 113 items, both rated on a three-point Likert scale (0 = not true to 2 = very true or often true). Both questionnaires consist of two broadband syndrome scales (Internalizing Problems and Externalizing Problems) and a Total Problems scale that consists of eight narrowband syndrome scales (Anxious/Depressed, Withdrawn/Depressed, Somatic Complaints, Aggressive Behaviour, Rule-Breaking Behaviour, Thought Problems, Social Problems, Attention Problems). Considering the heterogeneous character of the children’s diagnoses, Total Problems and the two broadband syndrome scales were used as an outcome variable in this study. Additionally, the narrowband syndrome scale Attention Problems was used as an outcome variable as almost a quarter of the children were diagnosed with ADHD and because the scale Attention Problems is not included in one of the two broadband scales (Achenbach and Rescorla 2001). Example questions of the ASR are ‘I cry a lot’ and ‘I am impulsive or act without thinking’, and of the CBCL are ‘Argues a lot’ and ‘Feels worthless or inferior’. Internal reliability for the ASR and CBCL was found to be excellent at pre-test, post-test and follow-up; Cronbach’s alpha for the ASR was .95, .94 and .97 respectively and for the CBCL .95, .96 and .96.

### Data Analyses

According to the Little’s MCAR test, data were missing at random. Results of the Kolmogorov–Smirnov test for the assumption of normality showed statistical significant results in a few variables, indicating a non-normal distribution for pre-test of IM-P (*D*(70) = .11, *p* < .05), pre-test of FFMQ (*D*(70) = .11, *p* < .05), pre-test of ASR (*D*(58) = .12, *p* < .05), and pre-test and post-test of CBCL (respectively *D*(54) = .15, *p* < .01, and *D*(37) = .19, *p* < .01). Evaluation of univariate outliers showed no outliers, using standardised values and a critical standardised score of ±3.29. For an overview of the means and standard deviations of the variables, see Table 1.

**Table 1:** Means and standard deviations of all variables at pre-test, post-test, and 8-week follow-up

	Pre-test		Post-test		Follow-up	
	*M*	*SD*	*M*	*SD*	*M*	*SD*
CBCL total	63.87	13.26	62.86	13.52	60.98	11.77
Internalising	63.44	12.62	60.19	14.84	59.73	12.36
Externalising	61.17	13.61	61.11	12.30	57.86	12.11
Attention	64.65	10.89	63.84	9.08	61.39	9.36
ASR total	59.02	11.19	54.24	10.06	51.58	12.89
Internalising	62.67	12.22	57.15	10.13	55.04	12.49
Externalising	57.28	9.11	53.59	10.15	50.72	12.15
Attention	62.50	9.89	60.41	9.39	58.15	8.86
IM-P total	3.15	.42	3.50	.43	3.63	.42
FFMQ total	2.99	.47	3.35	.39	3.51	.49
Observing	3.57	.89	3.84	.62	3.91	.68
Describing	3.28	.83	3.62	.57	3.66	.67
Awareness	2.77	.76	3.12	.58	3.33	.62
Non-judging	2.85	.71	3.14	.67	3.44	.84
Non-reactivity	2.62	.62	3.18	.54	3.24	.55

*M* mean, *SD* standard deviation, *CBCL* Child Behavior Checklist, *ASR* Adult Self Report, *FFMQ* Five Facets Mindfulness Questionnaire, *IM-P* Interpersonal Mindfulness in Parenting scale

CBCL and ASR are *t* scores; IM-P and FFMQ are mean item scores; the scale ranges were for CBCL and ASR 0–2 and for IM-P and FFMQ 1–5

Testing the first and second hypothesis, the effectiveness of the training, data were analysed using multilevel modelling (mixed models) in SPSS to incorporate all available data. However, as seven participants did not fill out the ASR at any measurement occasion, because some informant groups were not provided with all questionnaires at all measurement occasions, the analyses for the ASR were performed on data of 64 parents. The analyses for the CBCL were performed on data of 61 parents for two reasons. First, two parents who filled out the CBCL for ages 1.5–5 on one measurement and the CBCL for ages 6–18 on the next were not taken into account as these measures are not sufficiently comparable, and second, because seven parents did not fill out the CBCL at any measurement occasion. Taking into account gender and age of the parents and children, for the raw scores on the CBCL and the ASR, standardised *t* scores were calculated. For the variables IM-P, FFMQ, ASR, and CBCL, *z* scores were calculated to obtain more interpretable results, as the value zero now represents a mean score on these variables. Moreover, parameter estimates can now be interpreted similarly to Cohen’s *d* effect sizes.

## Results

### Direct and Delayed Effects of the Mindful Parenting Training on Mindfulness and Psychopathology

Regarding the first hypothesis, directly after the training, parents’ report showed a significant decline on the ASR in their total psychopathology symptoms, of medium effect size. Post hoc analyses of the three ASR syndrome scales showed a significant decline in parental internalising problems of medium effect size and a significant decline in their externalising and attention problems of small effect size. Parents’ report showed a significant decline on their children’s total psychopathology symptoms directly after the training on the CBCL, of small effect size. Post hoc analyses revealed a significant decline directly after the training in the three syndrome scales of the CBCL, internalising, externalising, and attention problems, of small effect size. The decline in parents’ psychopathology symptoms was maintained at follow-up; effect sizes of change from pre-test to follow-up were medium. For children’s psychopathology symptoms, the decline was also maintained, with small to medium effect sizes of change from pre-test to follow-up. Results of these multilevel data analyses are outlined in Table 2.

**Table 2:** Standardised parameter estimates of multilevel models of psychopathology outcome predicted by measurement occasion

	Pre–post		Pre–follow-up		Post–follow-up	
	*Par. Est.*	*SE*	*Par. Est.*	*SE*	*Par. Est.*	*SE*
ASR total	−.38**	.12	−.63***	.12	−.23	.16
Internalising	−.39**	.12	−.62***	.14	−.22	.16
Externalising	−.35**	.12	−.56***	.14	−.21	.13
Attention	−.21*	.10	−.44***	.10	−.23	.12
CBCL Total	−.25*	.10	−.33**	.10	−.07	.11
Internalising	−.34**	.10	−.31**	.10	.03	.12
Externalising	−.22*	.10	−.37**	.11	−.15	.10
Attention	−.26*	.10	−.42***	.11	−.17	.09

*ASR* Adult Self Report, *CBCL* Child Behavior Checklist, *Par. Est*. parameter estimates which can be interpreted as Cohen’s *d* effect sizes of change

*p < .05; **p < .01; ***p < .001

Regarding the second hypothesis, a significant increase in mindful parenting (IM-P total score) was reported by parents directly after the training and at follow-up, of respectively medium and large effect size. Compared to post-test, IM-P further improved significantly at follow-up, of small effect size. Furthermore, parents significantly improved on general mindful awareness (FFMQ total score) after the training and at follow-up as compared to before; effect sizes were respectively medium and large. Post hoc analyses reflected improvement in all five FFMQ subscales: Observing, Describing, Awareness, Non-judging, and Non-reactivity. Hence, they seemed to be better able to observe their inner experiences, to label sensations and thoughts with words, to focus their attention to their current activity, to evaluate thoughts and feelings objectively and unbiased, and to notice these thoughts and feelings without showing an immediate response. At follow-up, the total score on general mindful awareness was further significantly increased compared to post-test, of small effect size. Results of these multilevel data analyses are outlined in Table 3.

**Table 3:** Standardised parameter estimates of multilevel models of mindfulness outcome predicted by measurement occasion

	Pre–post		Pre–follow-up		Post–follow-up	
	*Par. Est.*	*SE*	*Par. Est.*	*SE*	*Par. Est.*	*SE*
IM-P total	.78***	.12	1.04***	.12	.25*	.12
FFMQ total	.73***	.12	1.02***	.13	.29**	.10
Observing	.33*	.14	.42**	.14	.10	.10
Describing	.44***	.09	.56***	.12	.12	.08
Awareness	.54***	.13	.78***	.14	.24*	.10
Non-judging	.37**	.13	.73***	.15	.36**	.13
Non-reactivity	.88***	.14	.98***	.12	.10	.10

*FFMQ* Five Facets Mindfulness Questionnaire, *IM-P* Interpersonal Mindfulness in Parenting scale, *Par. Est*. parameter estimates which can be interpreted as Cohen’s *d* effect sizes of change

*p < .05; **p < .01; ***p < .001

### Multicentre Results

The factor ‘location’ was added to the multilevel models to determine possible differences in treatment effects between mental health care clinics. Neither a main effect for location for IM-P and FFMQ, nor for ASR total problems occurred. However, a main effect for location was found for CBCL total problems (*F*(2, 57) = 11.02, *p* < .001). It was found that parents’ ratings of child psychopathology of UvA minds and Accare were lower across measurements compared to Dimence; however, all three mental health care clinics showed a similar decline in child psychopathology across measurement occasions. Therefore, differences in severity are not considered as problematic for the interpretation and generalisability of the results.

### Predicting Psychopathology Outcome in Parent and Child from General Mindfulness and Mindful Parenting

The third hypothesis was also tested using multilevel modelling. IM-P and FFMQ scores were aggregated, so the average score of one person is filled in on every measurement occasion. Then, scores of change for the IM-P and the FFMQ were calculated by subtracting the aggregated IM-P and FFMQ scores from the standardised IM-P and FFMQ scores. The aggregated scores represent the differences between individual persons, leaving out the treatment effects. The scores of change, therefore, represent the effects of the training within individual persons. Between the aggregated IM-P scores and the IM-P scores of change, the correlation is zero, so is the correlation between the aggregated FFMQ scores and the FFMQ scores of change. This indicates that effects within and between individual persons are completely separated, which makes it possible to determine whether an increase in mindfulness by following the training predicts a decline in parents’ and children’s psychopathology. The aggregated scores and the scores of change of both variables were standardised to obtain parameter estimates (*B*) that can be interpreted similarly to *r*-effect sizes. As the FFMQ and the IM-P are both measures of mindfulness and therefore measure partially the same construct, the correlation between the FFMQ and the IM-P was calculated. Results showed that the overall correlation between the FFMQ and the IM-P was significant and substantial (*r*(185) = .64, *p* < .001). Therefore, FFMQ and IM-P were both put into the model, to examine the effect of one variable corrected for the effect of the other variable.

### Parents Psychopathology Outcome

Results showed that an increase in FFMQ, corrected for the effect of IM-P, predicted a significant reduction in parental total psychopathology symptoms across measurements (*B* = −.19, *t*(64) = −2.52, *p* < .05), of small effect size. In this model, an increase in IM-P, corrected for the effect of FFMQ, did not predict a significant reduction in parental total psychopathology symptoms (*B* = −.12, *t*(54) = −1.58, *p* > .05). Post hoc analyses of the three ASR syndrome scales yielded similar results as an increase in FFMQ, corrected for the effect of IM-P, predicted a significant reduction in parental internalising and attention problems across measurements (respectively *B* = −.20, *t*(80) = −2.39, *p* < .05; *B* = −.19, *t*(65) = −3.17, *p* < .01), of small effect size. In turn, changes in IM-P, corrected for the effect of FFMQ, did not predict changes in parental internalising and attention problems (respectively *B* = −.08, *t*(68) = −.98, *p* > .05; *B* = −.03, *t*(66) = −.55, *p* > .05). Furthermore, a reduction in parental externalising behaviour problems was predicted neither by an increase in FFMQ nor by an increase in IM-P (respectively *B* = −.07, *t*(68) = −1.03, *p* > .05; *B* = −.11, *t*(79) = −1.58, *p* > .05).

### Children’s Psychopathology Outcome

Looking at the results for the FFMQ and IM-P, children’s total psychopathology symptoms over time were not affected by their parents’ increase in mindful parenting (respectively *B* = −.05, *t*(52) = −.90, *p* > .05; *B* = −.03, *t*(72) = −.51, *p* > .05). However, post hoc analyses of the three CBCL syndrome scales show that a significant reduction in children’s externalising problems, controlled for the effect of FFMQ, was predicted by an increase in IM-P (*B* = −.11, *t*(74) = −2.00, *p* < .05), of small effect size. An increase in FFMQ, however, did not predict a decrease in children’s externalising problems (*B* = −.04, *t*(60) = −.62, *p* > .05). Furthermore, increases in FFMQ and IM-P were not found to predict reductions in children’s internalising problems (respectively *B* = −.02, *t*(64) = −.26, *p* > .05; *B* = −.07, *t*(69) = −1.21, *p* > .05) nor in attention problems (respectively *B* = −.09, *t*(63) = −1.40, *p* > .05; *B* = .05, *t*(68) = .92, *p* > .05).

## Discussion

This study aimed to give a more fine-grained picture of the direct and delayed effects of a single mindful parenting training in a clinical setting on child and parental psychopathology and, building on this, to evaluate whether mindful parenting and general mindful awareness in parents are predictors for child and parental psychopathology outcomes.

With respect to the first hypothesis that parents’ and their children’s psychopathology symptoms decrease after the training, findings of the study of Bögels et al. (2014) were replicated as parents’ report showed that their own and their child’s psychopathology symptoms were considerably less severe after the training, both on post-test and follow-up. It seems remarkable that child psychopathology reduced as an indirect result of the relatively short mindful parenting training that only their parents attended, especially taking into account the heterogeneous character of the children as they were diagnosed with a wide range of psychiatric problems and were, therefore, not assessed on specific problem behaviour. Instead, they were measured on overall psychopathology, while questionnaires measuring target symptoms are more sensitive to change (Brown et al. 2001). However, the studies of Neece (2014), Srivastava et al. (2011) and Singh and colleagues (2006, 2007, 2014) who also included children with various behavioural problems found a reduction in child behavioural problems (e.g. attention problems and aggressive and anxious behaviour) as well.

Furthermore, it is interesting that psychopathology was reduced not only in children but also in parents themselves, given the fact that most parents were referred to one of the mental health care clinics because of their child’s diagnosis rather than their own. Effects of the training were even stronger for changes in parental psychopathology than for changes in psychopathology of their children. However, these results are in line with the studies of Bögels et al. (2014), Dykens et al. (2014) and Neece (2014), who also found a decline in parental psychopathology after the mindfulness-based parenting training.

In concordance with the second hypothesis that mindful parenting and general mindful awareness in parents increase after participation in mindful parenting training, findings indicate that parents see themselves as more mindful in general after the training and as more mindful in their parenting, both of medium effect size. At follow-up, general mindful awareness and mindful parenting further improved. These results are in line with the study of Ferraioli and Harris (2013) and the two non-clinical studies of Coatsworth et al. (2010) and Altmaier and Maloney (2007), who also found positive effects of a mindfulness-based parenting program on measures of mindful awareness and mindful parenting.

The third hypothesis, that change in parents’ general mindful awareness and mindful parenting predicts a decline in child and parental psychopathology by following the mindful parenting training, was supported for parents as improvements in parental general mindful awareness predicted a reduction in parental psychopathology. Changes in mindful parenting, however, did not predict changes in parental psychopathology. An explanation might be that mindful parenting is not initially meant to make changes in parents’ own psychopathology symptoms, rather to make changes in the interaction with their child, whereas general mindfulness is thought of as a way to cope with struggles in a participants’ own life. To the scope of our knowledge, this is the first time that change in mindfulness itself is found to predict change in psychopathology in parents following mindful parenting training. As for children, only improved mindful parenting, but not general mindfulness, is found to significantly reduce externalising problems of children as reported by their parents.

It makes sense that only increase in mindful parenting, and not in general mindfulness, accounts for changes in child psychopathology, as it can be expected that only when parents become more mindful in (challenging) parenting situations, child behaviour problems will lessen. Moreover, it would be interesting to investigate in further research if parental general mindful awareness and mindful parenting do also account for changes in other outcome variables. For example, in the non-clinical study of Coatsworth et al. (2010), it was found that effects of the mindful parenting training indirectly improved the parent–youth relationship through changes in mindful parenting.

A limitation to this study is that the data is correlational; therefore, causal inferences of the third hypothesis cannot be made. The significant results of testing this hypothesis are only evidence supporting the hypothesis but do not allow determining whether the direction of the hypothesis is true. To test causality, it is, therefore, recommended for future research to use data from a randomised controlled trial. The decision to test whether mindfulness predicts psychopathology, and not vice versa, is because mindfulness, and not psychopathology, is actively manipulated during the mindfulness sessions. Moreover, the third hypothesis is theoretically supported by the studies that were included in the meta-analytic review of Gu et al. (2015) and in the systematic review of Van der Velden et al. (2015), as mindfulness was found to be a mechanism of change for psychopathology in adults following mindfulness-based therapy.

Another limitation is that measurement occasions in this study only took place at pre-test, post-test and follow-up, whereas weekly measurements would have allowed this study to give a more detailed picture of when change in mindfulness, mindful parenting and psychopathology starts to change. Furthermore, it was only during this study that a more extended version of the IM-P became available for the Dutch population (De Bruin et al. 2014), and could therefore not yet be incorporated in this study. This extended version, however, has a higher reliability than the short version and contains different subscales and is recommended for use in future studies. This study included an experimental group, but no (waitlist) control group or active control intervention. It is recommended that future research is based on a randomised control trial in which mindful parenting training is compared to a proven effective parenting training (e.g. parent management training) and a control group. In addition, it would be interesting to conduct a similar study evaluating the effects of mindful parenting training as a stand-alone intervention versus a mindful parenting training with a parallel mindfulness training for children, similar to the studies of Van der Oord et al. (2012) and Van de Weijer-Bergsma et al. (2011). A combined training may be more effective, on measures of child psychopathology, than mindful parenting training on its own (Harnett and Dawe 2012).

Moreover, this study did not include informants other than the parents participating in the training. In order to gain more objective results, it is recommended that informants who are involved in the parent’s and child’s environment on a daily basis (e.g. teachers or the non-participating parent) and children themselves are also given the opportunity to report about (changes in) mindfulness and mindful parenting of the participating parent and parental and children’s psychopathological symptoms. Furthermore, it would be interesting for future studies to include clinician ratings and observational measures of the child behaviour. In the non-clinical study of Coatsworth et al. (2010), the children of participating mothers were asked to complete measures on mothers’ discipline consistency, monitoring, anger management, and their relationship with their mother. Children of parents in the mindfulness-based parenting group did not report differently on these measures than children of mothers in the control group. Furthermore, long-term follow-ups are required in order to assess whether the positive effects of the training are maintained or even further increase over a longer period of time.

This study contributes to the burgeoning work in the field of mindful parenting. A strength of this study is the multicentre design in a ‘real-world’ effectiveness trial. As this study took place in three mental health care clinics, positive outcomes of mindful parenting training appear to be generalisable to other centres than the one where this training was developed. As this study only included parents referred to secondary mental health care clinics, findings can be generalised to clinical practice and are relevant for other parents in need for treatment. All trainers followed a standardised protocol and parents were given the same manual, making this study replicable. Another strength is the assessment of various aspects of general mindfulness and of mindful parenting. A final strength of this study is the inclusion of a relatively large sample size and the measurement of child psychopathology, parental psychopathology, mindfulness, and mindful parenting with well-validated measures.

Mindful parenting is a relative new intervention that appears to be effective for parents with children suffering from psychopathology. The low drop-out rate, participation of multiple centres, strong motivation of participants to practice, large number of participants that can participate on one group, and the continued maintenance of improvement after the intervention had ended are all indicators that mindful parenting is an acceptable and a feasible intervention in mental health care. Also, the heterogeneity of the groups, in terms of child age and child and parental problems, contributes to the feasibility of the intervention, as the larger the variety of the parents that can participate in the training, the easier groups can be filled. As parents’ increased mindful parenting but not increased general mindfulness is found to predict child psychopathology, mindful parenting training rather than general mindfulness training appears to be the training of choice for parents of children with psychopathology. However, RCTs comparing mindful parenting to general mindfulness training and to parent management training are needed in order to shed more light on the effects of mindful parenting and its mechanisms of change.
